# Porous Microgels for Delivery of Curcumin: Microfluidics-Based Fabrication and Cytotoxicity Evaluation

**DOI:** 10.3390/mi14101969

**Published:** 2023-10-22

**Authors:** Sinem Orbay, Rana Sanyal, Amitav Sanyal

**Affiliations:** 1 Institute of Biomedical Engineering, Bogazici University, Istanbul 34684, Türkiye; sinem.orbay@boun.edu.tr; 2 Biomedical Engineering Department, Erzincan Binali Yildirim University, Erzincan 24002, Türkiye; 3 Department of Chemistry, Bogazici University, Istanbul 34342, Türkiye; 4 Center for Life Sciences and Technologies, Bogazici University, Istanbul 34342, Türkiye

**Keywords:** microfluidics, droplet-based microfluidics, polymeric microgels, drug delivery

## Abstract

Polymeric microgels, fabricated via microfluidic techniques, have garnered significant interest as versatile drug delivery carriers. Despite the advances, the loading and release of hydrophobic drugs such as curcumin from polymeric microgels is not trivial. Herein, we report that effective drug loading can be achieved by the design of porous particles and the use of supramolecular cyclodextrin-based curcumin complexes. The fabrication of porous microgels through the judicious choice of chemical precursors under flow conditions was established. The evaluation of the curcumin loading dependence on the porosity of the microgels was performed. Microgels with higher porosity exhibited better curcumin loading compared to those with lower porosity. Curcumin-loaded microgels released the drug, which, upon internalization by U87 MG human glioma cancer cells, induced cytotoxicity. The findings reported here provide valuable insights for the development of tailored drug delivery systems using a microfluidics-based platform and outline a strategy for the effective delivery of hydrophobic therapeutic agents such as curcumin through supramolecular complexation.

## 1. Introduction

Polymeric microgels, produced via the microfluidics technique, have emerged as a fascinating area of research in materials science and biotechnology [1,2,3]. These miniature hydrogel particles, typically ranging from hundreds of nanometers to a few micrometers in size, impart unique properties that make them vital in a wide range of applications [4,5,6]. Microfluidics, a technology that manipulates fluids in microscale channels, allows precise control over the synthesis process, resulting in well-defined and uniform polymeric microgels [7]. Fabricating microgels using microfluidics offers numerous advantages compared to traditional methods. The precise control over size and shape enabled by microfluidic devices ensures the production of monodisperse microgels, providing uniformity in their properties. Microfluidics devices enable high-throughput production, allowing the rapid generation of large quantities in a short time, thus facilitating scalability for mass production [8]. Moreover, the tunable properties of microgels, such as porosity and drug release kinetics, can be finely adjusted by manipulating the fabrication parameters. With reduced reagent consumption, ease of automation, and the seamless integration of other functionalities, microfluidics-fabricated microgels emerge as a versatile and cost-effective solution to advance biomedical research and enable cutting-edge technologies [9,10]. 

Due to their versatile and multifunctional nature, soft materials like hydrogels [11,12,13], microgels [14,15,16], and nanogels [17,18,19] are finding increasing use in applications such as drug delivery and diagnostics. Microgels are being explored as promising candidates for drug delivery systems due to their remarkable features, such as substantial loading capacity, compatibility with biological systems, and biostability [4,20,21,22]. The crosslinked network of microgels plays a crucial role in preventing the breakdown of long molecular chains, thereby contributing to its stability [23]. The viscoelastic characteristics of microgels add to their appeal by demonstrating structural changes in response to environmental changes. Additionally, stimuli-responsive microgels [24,25] exhibit a faster response to stimuli than bulk gels, proving their efficiency in drug delivery systems [26,27,28,29]. Another aspect is that modulating the porosity of microgels through precise adjustments in monomer compositions has a significant influence on drug delivery. By tailoring the monomer constituents, it becomes possible to engineer microgels with varying degrees of porosity. This, in turn, directly impacts the performance and efficacy of the drug delivery process.

The fabrication of microgels with a porous morphology enables control over the diffusion of drugs from within the gel matrix [30,31]. Microgels with higher porosity exhibit larger interstitial spaces, facilitating the faster diffusion and release of encapsulated drugs. Moreover, the porosity of microgels influences their loading capacity. Microgels with increased porosity can accommodate a larger quantity of drugs, enhancing the total payload that they can deliver. This is particularly valuable when aiming to administer therapeutic agents in precise amounts over extended periods. Additionally, porosity impacts the interaction between microgels and their environment. Microgels with higher porosity tend to be more responsive to external stimuli [32], leading to more pronounced changes in their size and structure. This responsiveness can be harnessed to trigger accelerated drug release when desired, providing an on-demand delivery mechanism.

Curcumin, a naturally occurring polyphenolic compound sourced from the rhizome of turmeric (Curcuma longa), exhibits a wide array of biological and health-enhancing attributes. These encompass antioxidant, antimicrobial, anti-inflammatory, anti-proliferative, and anti-cancer activities, making it a versatile and valuable compound [33,34,35]. However, its potential for clinical applications remains limited due to curcumin’s challenges in terms of low bioavailability, limited absorption, aqueous solubility, and rapid metabolism. Therefore, curcumin has been explored using a variety of delivery techniques. These include liposomes [36], micelles [27,37], lipid-based nanoparticles [38], and polymer–drug conjugates [39], often involving physical encapsulation or chemical conjugation for its incorporation. Curcumin beta-cyclodextrin inclusion complexes represent a solution to the inherent limitations of curcumin, including poor solubility, low bioavailability, and susceptibility to degradation. Through the complexation of curcumin with beta-cyclodextrin, its solubility is significantly improved, ensuring better absorption in the body and increased bioavailability [40,41]. Furthermore, these complexes play a pivotal role in bolstering curcumin’s stability, shielding it from detrimental environmental factors like light, heat, and oxygen, which can cause degradation. Additionally, the capacity to regulate release rates enables the sustained and controlled delivery of curcumin over time [42]. Therefore, curcumin beta-cyclodextrin inclusion complexes offer a versatile and potent solution to unlock the therapeutic potential of curcumin.

**Figure 1:**
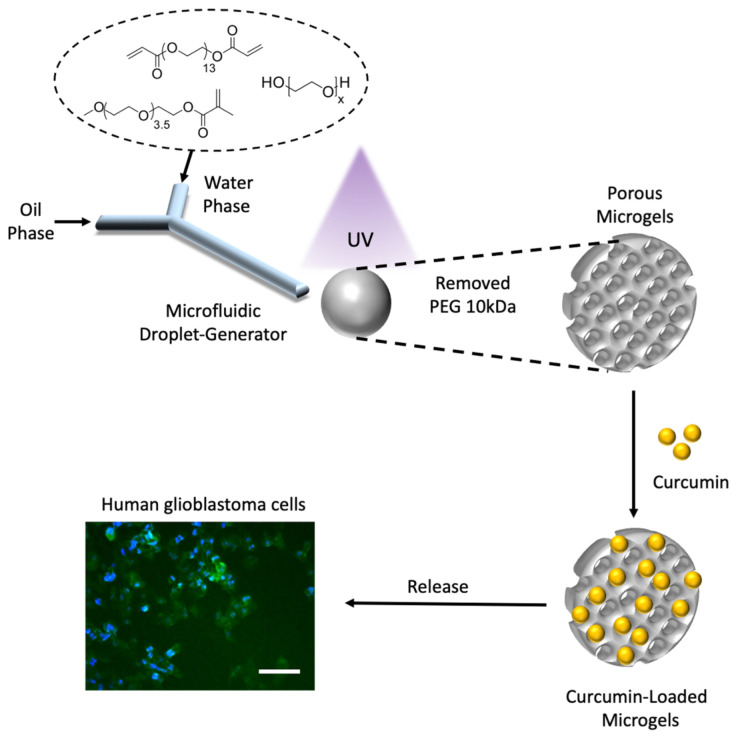
Schematic illustration of microfluidics-based fabrication of porous microgels for effective delivery of curcumin to cancer cells (scale 100 μm).

Herein, we fabricate non-porous and porous microgels using a microfluidics platform and demonstrate the effective loading of a curcumin cyclodextrin complex into these microgels (Figure 1). We show that the fine tuning of the monomer composition and porosity has a profound effect on the efficiency of these microgels as drug delivery vehicles. This study delves into the intricate relationship between microgel morphologies, drug encapsulation and release, and cytotoxicity, thus providing valuable insights into the future of the microfluidics-based tailoring of drug delivery systems.

## 2. Materials and Methods

### 2.1. Materials and Devices

All solvents were purchased from Merck and used as obtained without further purification. Ultrapure water was obtained from a Milli-Q Water Purification System (Millipore, Billerica, MA, USA). Polyethylene glycol diacrylate (PEGDA), poly(ethylene glycol) methyl ether methacrylate (PEGMEMA), poly(ethylene glycol) 10kDA, 2,2-diphenyl-1-picrylhydrazyl (DPPH), lithium phenyl-2,4,6-trimethylbenzoylphosphinate (LAP), (2-hydroxypropyl)-β-cyclodextrin (HP-*β*-CD), and curcumin were obtained from Sigma Aldrich (Sigma, St. Louis, MO, USA).

For the microfluidics setup, polydimethylsiloxane (PDMS), hexamethyldisilane (HMDS), chlorotrimethylsilane, a negative photoresist (SU-8 2040), propylene glycol monomethyl ether acetate (PGMEA), glass slides and a biopsy punch (Harris Uni-Core 0.75 mm, USA), and a nonionic surfactant (Span 80) were purchased from Sigma Aldrich. Mineral oil (0.880 kg/L) was purchased from AppliChem. Adhesion promoter TI prime was purchased from MicroChemicals. PTFE tubes for the inlets and outlets of microfluidic channels were obtained from Microfluidic ChipShop (Microfluidic ChipShop, Jena, Germany).

An EVG 620 semiautomatic contact photolithographic alignment and exposure tool with a 1000-watt light source was used for the silicon master mold fabrication (EV Group, Sankt Florian am Inn, Austria). Plasma treatment of the PDMS device and a glass slide was performed using a laboratory corona treater (BD-20AC, Electro-Technic Products Inc., Chicago, IL, USA).

In situ photopolymerization was performed using a UV light source (36 W, 365 nm). UV-vis spectra were collected on a Cary Varian spectrometer (Santa Clara, CA, USA). Curcumin-loaded polymeric microgels were visualized using fluorescence microscopy (HBO100 ZEISS Fluorescence Microscopy, Carl Zeiss., Jena, Germany), and fluorescence images were processed using the Zeiss AxioVision software. Syringe pumps were purchased from maviTeknik (Mersin, Turkiye).

Cell viability experiments were performed with a plate reader (Multiscan FC, Thermo Scientific, Waltham, MA, USA) using Cell Counting Kit-8 (CCK-8, Fluka, Sigma, St. Louis, MO, USA). Images of drug internalized cells were obtained using a Zeiss Observer Z1 fluorescence microscope with an Axiocam MRc5 camera. The U87 MG cell line was purchased from ATCC (LGC Standards, Wesel, Germany) and grown according to the culture method requirements of the manufacturer. All the chemicals used in cell experiments were purchased from Sigma Aldrich.

### 2.2. Microfluidics Channel Fabrication

**Figure 2:**
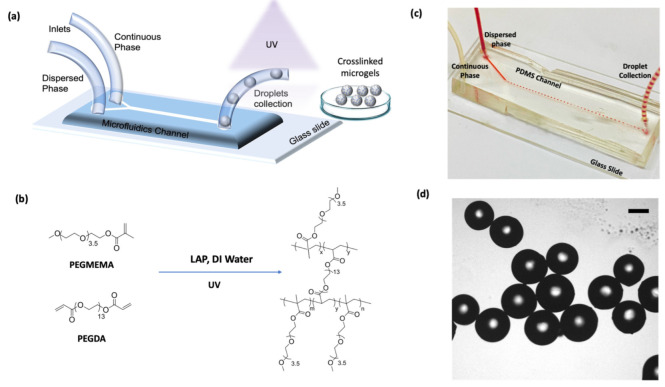
(**a**) General illustration of microfluidics fabrication of polymeric microgels, (**b**) chemical scheme of microgel formation, (**c**) real droplet generator in operation, (**d**) optical image of monodisperse microgels in dry state produced via microfluidics (scale 200 μm).

To create a single-layer microfluidic channel of polydimethylsiloxane (PDMS) with dimensions measuring 400 μm in width, 300 μm in depth, and 3 mm in length, as illustrated in Figure 2a, the initial step involved crafting a replication mold using a conventional lithography technique. To produce the master mold, a 4-inch silicon wafer underwent a meticulous cleaning process, followed by silane vapor treatment utilizing HMDS for one hour. After applying a coating of TI prime to serve as an adhesion promoter, a brief one-minute pre-bake was carried out on a hotplate at 95 °C. Subsequently, a layer of SU-8 2050 was spun onto the wafer and it was subjected to a baking process at 65 β °C for one minute, followed by 20 min at 95 β °C before the exposure stage.

The photomask, bearing the desired pattern, was positioned pattern side up on a 5 × 5-inch soda-lime glass substrate, which was then placed face down on a mask aligner. The silicon wafer coated with a photoresist was precisely aligned with the mask and brought into contact to enable exposure. After exposure, the wafer was post-baked at 65 °C for 5 min and 95 °C for 10 min. Subsequent immersion in an organic SU-8 developer solvent (PGMEA) for 10 min facilitated the removal of excess photoresist. The wafer underwent sequential rinsing with isopropanol and DI water, followed by nitrogen drying. To enable the reuse of the mold, the wafer’s surface was treated with chlorotrimethylsilane vapor within a vacuum desiccator.

The next phase Involved mixing a silicone elastomer base with a curing agent in a 10:1 ratio within a controlled environment devoid of dust. This mixture was then cured at 65 β °C for 2 h, leading to the replication of PDMS channels. Once complete curing was achieved, the PDMS was extracted from the master mold, and each microchannel was individually cut. The inlet and outlet points were established using a manually operated biopsy puncher. Both the PDMS channel and the glass slide surfaces underwent oxygen plasma treatment for 3 min to enhance bonding. The bonding itself was performed at 65 β °C for a night. Lastly, PTFE tubes were inserted into the inlets and outlets of the PDMS microfluidics channel in preparation for experiments.

### 2.3. Microgel Synthesis via Microfluidics

Microgels of varying porosity were fabricated using the water-in-oil (W/O) emulsion technique within a droplet-based microfluidic setup. A Y-shaped microfluidic channel was employed to generate consistently sized droplets (Figure 2a–d). The oil phase was pumped to create a continuous phase, and the aqueous phase, including the monomer solution and initiator, was injected into the channel to form a dispersed phase. The dispersed phase was then broken off when it met the continuous phase at the intersection of the channel. After microdroplet formation, the droplets traversed the channel and reached the outlet tubing, where UV polymerization was initiated. The microgels were collected in a container to undergo thorough washing steps to eliminate residuals, unreacted monomers, and PEG.

PEGDA, PEGMEMA, and PEG (10 kDa) were dissolved in DI water to prepare the dispersed phase. To catalyze the polymerization, a photoinitiator, LAP (constituting 2% of the total monomers), was introduced into this solution. This aqueous solution was then utilized as the dispersed phase in the microfluidics process, while the mineral oil with 10% SPAN 80 was employed as the continuous phase (Figure 2b).

### 2.4. Morphological Analysis Using Scanning Electron Microscopy (SEM)

Scanning electron microscopy (SEM) studies were conducted to characterize the microgels’ morphologies. SEM images were obtained from dried microgel samples using a JCM-5000 NeoScop Tabletop SEM with an accelerating voltage of 10 kV.

### 2.5. Curcumin and HP-β-CD Complexation

The complexation of curcumin and HP-*β*-CD was achieved through solvent evaporation methods, as described in a previous report [43,44]. Curcumin and HP-*β*-CD were dissolved in a 1:2 ratio in ethanol and stirred at 40 β °C until the ethanol completely evaporated. The resulting mixture was re-dissolved in water, filtered, and subjected to lyophilization. Subsequently, the sample was stored at 4 β °C for further analysis.

### 2.6. Curcumin Loading and Release Studies

Curcumin was loaded into the microgels using a solution absorption method. Microgels (~5 mg) were soaked in 1 mg/mL curcumin–HP-*β*-CD complex aqueous solution at 37 °C with 100 rpm mechanical shaking for 1 hr. The curcumin loading was monitored by analyzing the curcumin concentration in the soaking solution with a UV-vis spectrometer at 425 nm. Later, the curcumin-loaded microgels were gently washed with water, and 1 mL release medium was added. The release experiments were performed at 37 °C with 100 rpm shaking. At predetermined time intervals, the drug concentration in the collected media was monitored using a UV-vis spectrometer.

### 2.7. Drug Release Kinetic Models

Various kinetic models, including zero-order, first-order, Higuchi, and Korsmeyer–Peppas, were employed to validate and elucidate the underlying mechanism of drug release. To predict the release behavior of the drug, it is imperative to analyze diverse outcomes derived from fitting the data to a range of validated mathematical models [45,46]. These kinetic models elucidate whether the drug release is contingent on dissolution or diffusion. They also distinguish whether the release aligns with Fickian, non-Fickian, or super-case-II drug release models.

### 2.8. Antioxidant Activity

The antioxidant activity of the curcumin-loaded microgels was measured using the 2,2-diphenyl-1-picrylhydrazyl (DPPH) radical scavenging activity test. It involves exposing a purple-colored DPPH radical to a sample containing potential antioxidants. Antioxidants reduce the DPPH radical, causing it to change color from purple to yellow, which is quantified by measuring the decrease in absorbance at a specific wavelength using a UV spectrophotometer [47]. The extent of color change reflects the antioxidant capacity of the tested compound. To assess the antioxidant activity of the curcumin-loaded microgels, we prepared curcumin-loaded and non-loaded microgels. We measured the absorbance at 517 nm to demonstrate the inhibition of the DPPH radical.

### 2.9. In Vitro Cytotoxicity and Internalization

The cytotoxicity of drug-loaded microgels was determined using the CCK-8 viability assay. The U87 MG human glioma cell line (purchased from ATCC (LGC Standards, Wesel, Germany) was used for cytotoxicity experiments. Cells were grown in DMEM supplemented with 10% fetal bovine serum and incubated at 37 °C. U87 MG cells (4000 cells/well) were seeded in a 96-well plate in 100 μL DMEM and incubated at 37 °C for 24 h to adhere completely. Cell-adhered plates were treated with aliquots of drug-loaded microgels at 37 β °C for 24 h and 48 h. After 48 h, a CCK-8 solution (10%) was introduced into each well. Following a two-hour incubation period, the absorbance values were quantified using a plate reader (Multiscan FC, Thermo Scientific, Waltham, MA, USA) set at 450 nm. The cell viability of the treated cells was assessed based on the percentage relative to control cells (cells in media only). The results were analyzed using the GraphPad Prism software employing a nonlinear regression model. To facilitate cellular internalization, we initially seeded U-87 MG glioblastoma cells (50,000 cells per well) into a 24-well plate, triplicating the setup, with each well containing 1 mL of DMEM culture media. These cell cultures were then incubated at 37 β °C for 24 h. The cells were then subjected to three consecutive washes with 500 μL of PBS each time. Afterward, the cells were fixed by exposure to a 4% formaldehyde solution at 37 β °C for 10 min. Following another three rounds of PBS washing, the cells underwent 15-min incubation at 37 β °C for DAPI nuclei staining. We employed a Zeiss Observer Z1 fluorescence microscope to visualize and document the cellular state and processed the obtained images using the AxioVision software (Version 4.8.2.0).

## 3. Results

### 3.1. Microgel Synthesis and Characterization

Through a photo-initiated polymerization process using PEGDA, PEGMEMA, and PEG (10 kDa), polymeric microgels were successfully synthesized using microfluidics, as illustrated in Figure 2d. The gelation was accomplished under UV irradiation at 365 nm in the presence of the photoinitiator LAP. This approach harnessed the precise manipulation of fluid dynamics within microchannels by adjusting the flow rates, forming monodispersed microdroplets.

The dispersed phase consisted of PEG monomers, which were delivered by one of the inlets, as summarized in Table 1. The continuous phase consisted of 10 wt% Span 80. All components were pumped into the microfluidic channel via tubing attached to the tips of 1 mL syringes. Pumping into the channel was achieved using a syringe pump at a flow rate of 10 µL/min for the continuous phase and 5 µL/min for the dispersed phase. Droplet formation was observed once a steady flow of both phases had been established. These microdroplets underwent photopolymerization while traversing the outlet tubing.

**Figure 3:**
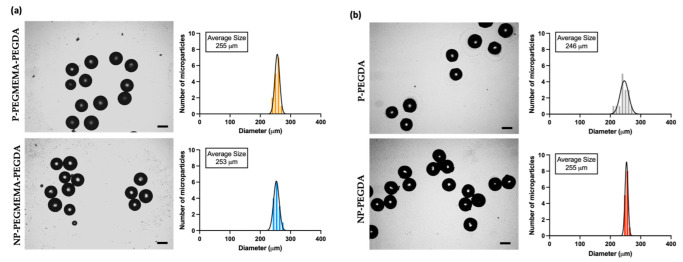
Size distribution of microgels. (**a**) Microgels with PEGMEMA, (**b**) microgels without PEGMEMA (scale 200 μm).

**Figure 4:**
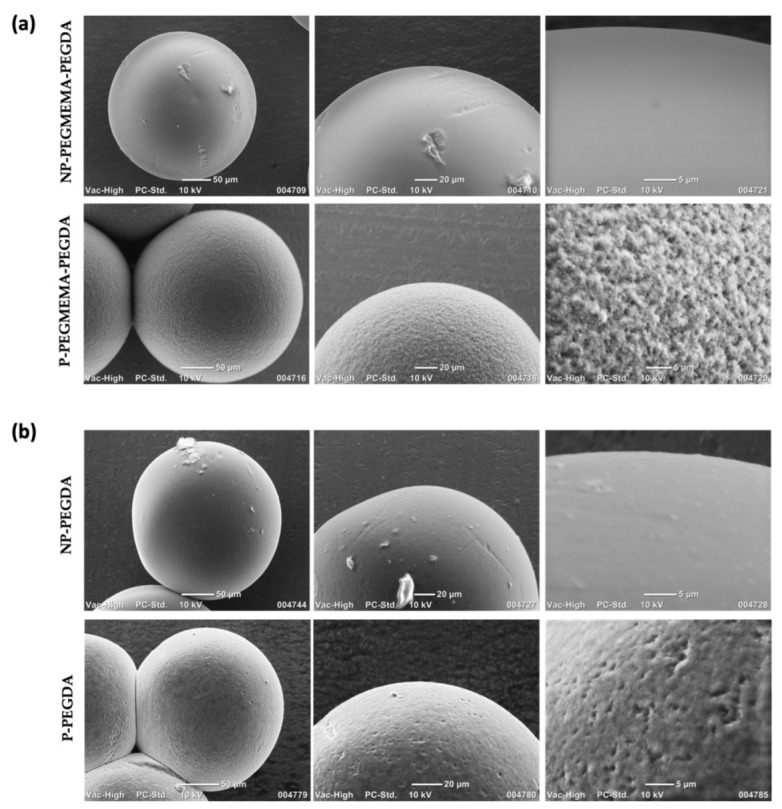
SEM images of microgels. (**a**) Microgels with PEGMEMA, (**b**) microgels without PEGMEMA.

After microgel formation, the microgels were collected into an Eppendorf tube. The microgels were washed with hexane, methanol, DI water, and methanol, respectively, to remove excess oil. Then, the microgels were dried overnight at room temperature under a vacuum. Thus, the synthesized microgels were examined using an optical microscope, allowing for the acquisition of size distributions (Figure 3a,b). Thereafter, SEM was utilized to probe the surface microstructures of the microgels. The microgels were dried before the analysis. It is known that porogens, often PEG-based, can be introduced during fabrication to increase porosity [48,49,50]. From the SEM images, morphologies with increased pore size were observed upon adding the chain length of PEG 10 kDa (Figure 4). Additionally, we inferred that enhanced porosity was obtained upon the incorporation of PEGMEMA as the monomer, along with the linear PEG (10 kDa). The comparison of Figure 4a,b indicates that the utilization of PEGMEMA resulted in a significant augmentation in the microgels’ porosity. The impact of incorporating PEG (10 kDa) into the polymeric network was explored within both PEGMEMA-containing and PEGMEMA-free microgels to elucidate the effect of individual components in terms of porosity and drug-loading capacities.

**Figure 5:**
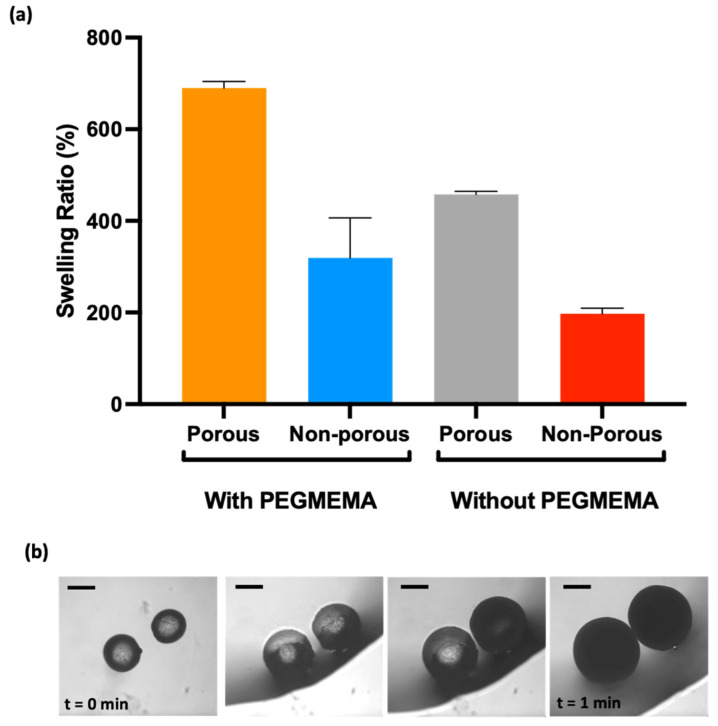
(**a**) Volume changes of microgels, (**b**) P-PEGMEMA-PEGDA microgel swelling profile video images (scale 200 μm).

To obtain further insights, water-induced swelling was employed to investigate the relationship between the volume change of dry microgels and their porosity. Dry microgels were first prepared and characterized for their initial size and structure. Then, a known amount of water was gradually introduced to the microgels, and their volume changes were meticulously recorded using an optical microscope. As water permeated the microgel network, the polymer chains within the microgels adsorbed water molecules, expanding the microgel structure. The extent of the volume change was directly proportional to the porosity of the microgels, as seen in Figure 5a, with more porous microgels (P-PEGMEMA-PEGDA) exhibiting larger volume increases due to the greater accessibility of water molecules to the internal void spaces. Figure 5b displays optical microscopy images capturing the swelling behavior of porous microgels upon the addition of 100 μL of water to the initially dry gels.

### 3.2. Curcumin Loading and Release Studies

**Figure 6:**
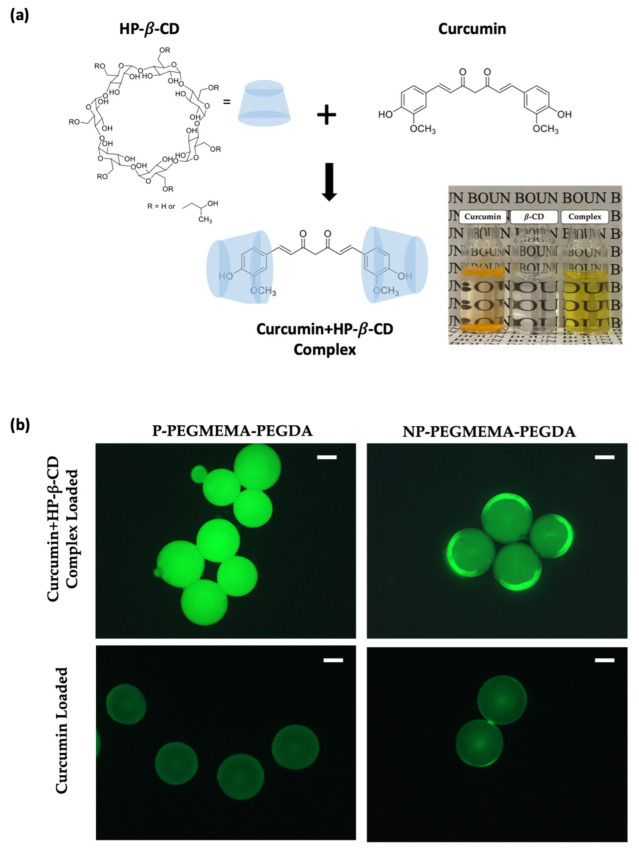
(**a**) Curcumin and β-cyclodextrin (HP-*β*-CD) complexation and solubility in DI water, (**b**) fluorescence microscopy images to compare the differences in only curcumin-loaded and curcumin–CD-complex-loaded microgels under the same exposure.

Microgels’ drug loading and release characteristics were examined using a hydrophobic drug, curcumin. The loading of the drug onto the microgel samples was accomplished through a solution absorption technique. Curcumin loading trials were conducted in deionized (DI) water, and, given curcumin’s hydrophobic nature, a curcumin–(2-hydroxypropyl)-*β*-cyclodextrin (HP-*β*-CD) complex was employed to enhance the drug’s solubility to obtain better loading, as seen in Figure 6a. Figure 6b demonstrates that using curcumin alone for loading, without CD complexation, did not yield effective results. This suggests that the supramolecular complex plays a crucial role in enhancing the loading performance, which will be important in obtaining sufficient release to induce cytotoxicity. An additional analysis was conducted to establish the formation of inclusion complexes between CUR and HP-*β*-CD through the analysis of FTIR (Figure S1) and ^1^H NMR (Figure S2) spectra.

**Figure 7:**
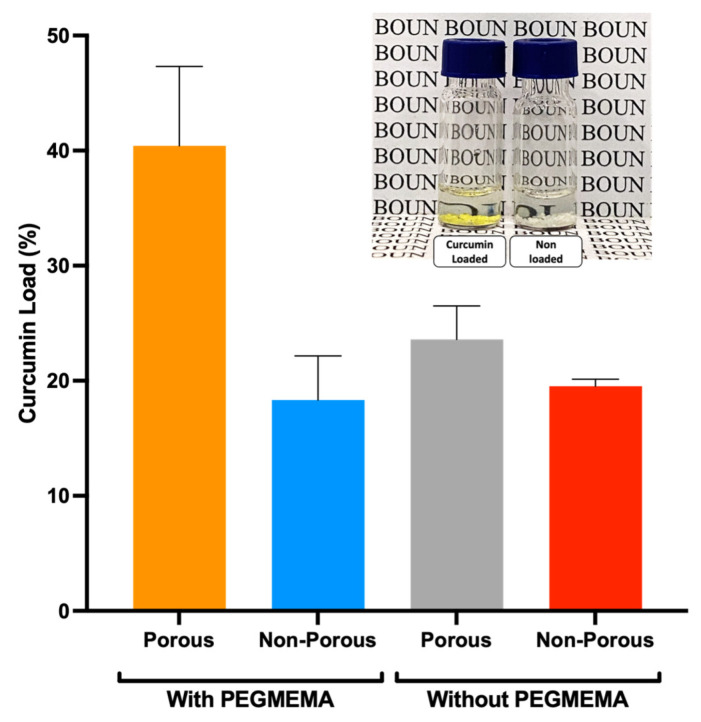
Comparison of curcumin loading in different microgel systems.

For loading studies, the curcumin–CD complex was dissolved in water (1 mg/mL). A predetermined amount of dry microgel was weighed and placed in separate vials for each microgel system. A calculated volume of the curcumin–CD solution was added to each vial, and the microgels were allowed to adsorb the complex at 37 °C with 100 rpm gentle mechanical shaking for 1 h. After adsorption, the microgels were separated from the solution, and the concentration of curcumin in the supernatant was measured using UV spectrophotometry at 425 nm. It enabled the calculation of the loaded curcumin by subtracting the initial concentration from the measured concentration. This loading process was repeated for microgels of varying porosity, and the data were analyzed to understand the influence of porosity on the curcumin adsorption efficiency, as drawn in Figure 7.

**Figure 8:**
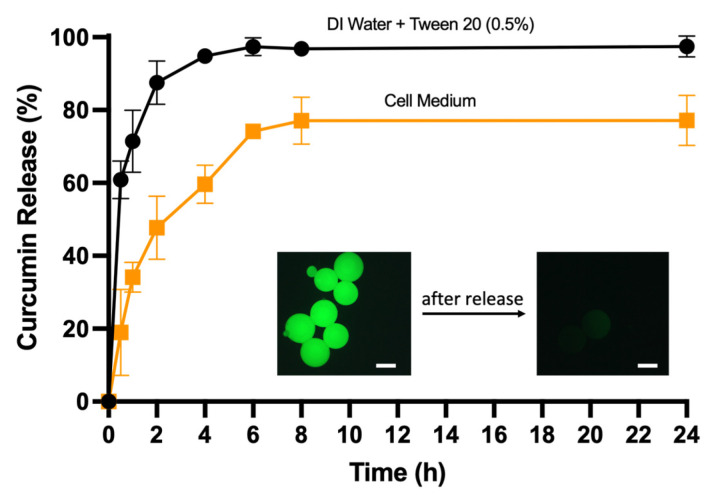
Curcumin release from P-PEGMEMA-PEGDA microgels in DI water + Tween20 (0.5%) and cell medium. Comparison of fluorescence microscopy images before and after drug release (scale 200 μm).

The optimal microgel system with the highest loading performance was employed to monitor the release profile of curcumin. The curcumin-loaded microgels were carefully washed with distilled water before introduction into the release medium. Release studies were conducted in both DI water with 0.5% Tween 20 and cell medium. At specific time intervals, the release medium was analyzed using a UV spectrophotometer to monitor drug release. The obtained release profiles demonstrated effective release in cell media, as also noted by the loss of fluorescence of the microgels upon drug release (Figure 8).

### 3.3. Drug Release Kinetic Models

The most suitable kinetic model to predict the release of curcumin from spherical microgels was determined through curve fitting and the calculation of correlation coefficients. These models help us to understand and predict how drugs are released over time, influencing their efficacy. Various models, such as zero-order (constant release rate), first-order (proportional to remaining drug), Higuchi (diffusion-based release), and Korsmeyer–Peppas (complex release mechanisms), were investigated for curcumin release by fitting the experimental data to these models using the following equations:

$$M_{t}/M=k_{0}t\;(\mathrm{Zero Order Model})$$

$$M_{t}/M=1-e^{-k_{1}t\;(\mathrm{First Order Model})}$$

$$M_{t}/M=k_{h}t^{0.5}\;(\mathrm{Higuchi Model})$$

$$M_{t}/M=kt^{n}\;(\mathrm{Korsmeyer-Peppas Model})$$

**Figure 9:**
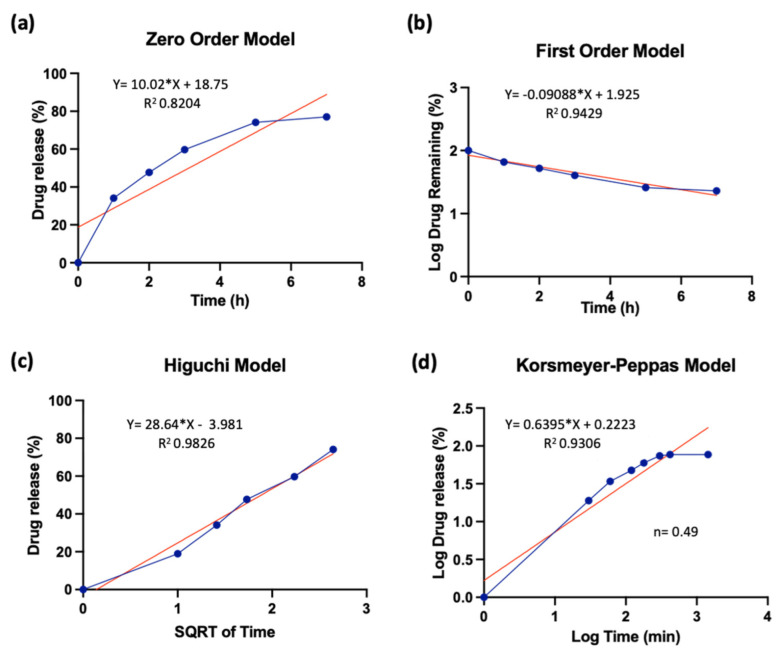
Model fitting of curcumin release kinetics. (**a**) Zero-order model, (**b**) first-order model, (**c**) Higuchi model, and (**d**) Korsmeyer–Peppas model.

The drug release dynamics of the composite hydrogel were investigated using the Korsmeyer–Peppas model, which characterizes drug release kinetics based on alterations in the diffusion coefficients of both water and the drug. The diffusion index (n) obtained from the Korsmeyer–Peppas model was 0.4857, indicative of non-Fickian (anomalous) diffusion in the range of 0.45 to 0.89. Anomalous transport encompasses a spectrum of behaviors that lie between simple Fickian diffusion and more intricate processes like erosion, swelling, relaxation, or chemical reactions. The fitting curves’ correlation coefficients for the release of curcumin from the spherical microgels were found to be 0.8204, 0.9429, 0.9826, and 0.9306, as seen in Figure 9. Among these, the Higuchi model exhibited the highest correlation coefficient. The Higuchi model had the highest correlation coefficient when fitting the release data of curcumin from the spherical microgels; this suggests that the release mechanism for curcumin from these microgels is consistent with diffusion-based release, where the amount released increases with the square root of time.

### 3.4. Antioxidant Activity of Curcumin-Loaded Microgels

**Figure 10:**
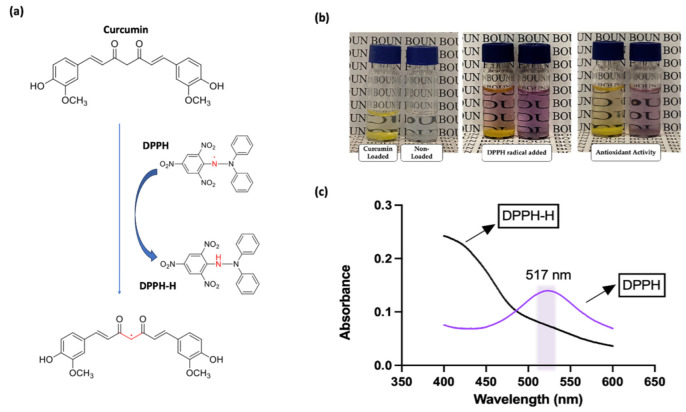
(**a**) Curcumin reaction with DPPH, (**b**) DPPH radical color change in the presence of curcumin, (**c**) DPPH radical scavenging UV spectroscopy result.

The DPPH radical scavenging activity of curcumin-loaded and non-loaded microgels was evaluated to assess their potential antioxidant properties. For the DPPH radical scavenging test, curcumin-loaded and non-loaded microgels were combined with 1 mL of an ethanolic DPPH solution (0.2 mmol/L). Subsequently, the resultant mixtures were left to stand for 30 min at room temperature in the dark (Figure 10b). The absorbance of the resulting supernatants was then measured spectrophotometrically at 517 nm. The DPPH radical is a stable free radical with a deep purple color in solution, and it changes color to yellow when it is reduced by an antioxidant compound, which can be observed in UV spectroscopy, as seen in Figure 10c. These results clearly indicate that incorporating curcumin into the microgels does not compromise its antioxidant activity. A slight delay in the quenching of the radical is observed for the curcumin-loaded microgels as compared to the free and complexed drug, presumably due to the slower release of the drug from the microgels (Figure S4).

### 3.5. In Vitro Studies

**Figure 11:**
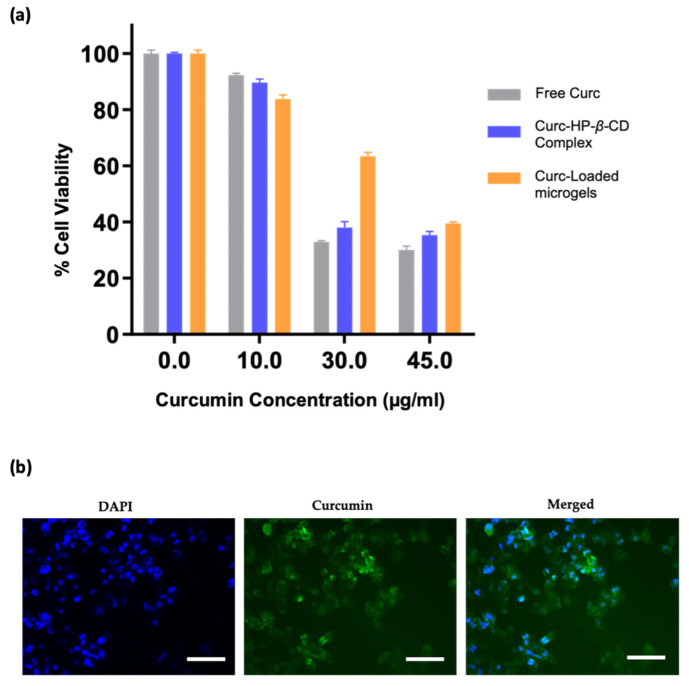
(**a**) Cell viability plot, (**b**) fluorescence images indicating the internalization of curcumin released from microgels, with blue (DAPI stain) to mark cell nuclei. Scale bar (100 μm).

This study aimed to design microgels to evaluate their potential as biomaterials for the delivery of the highly hydrophobic therapeutic agent curcumin. Hence, it was crucial to assess the cytotoxicity of these microgels and their resultant drug release products. Therefore, we evaluated the anti-cancer activity of curcumin-loaded microgels (P-PEGMEMA-PEGDA) against U-87 MG human glioblastoma cells by assessing their cytotoxicity using the CCK-8 assay. In Figure 11a, it can be observed that the cell viability decreased after a 48 h treatment with various curcumin concentrations. At the lowest concentration of 10 μg/mL, there was a modest reduction in cell viability, with approximately 85% of cells remaining viable. As the concentration of curcumin was increased to 30 μg/mL and 45 μg/mL, cell viability decreased progressively to 65% and 40%, respectively. These findings suggest that curcumin exerts a significant inhibitory effect on U87MG cell proliferation, highlighting its potential as a candidate for further investigation in the context of glioblastoma therapy.

We also investigated the cellular internalization of the drug using fluorescence microscopy, since curcumin is inherently fluorescent. Cancer cells were treated with aliquots from drug-loaded microgels for 6 h. Following incubation, we employed 4′,6-diamino-2-phenylindole (DAPI), a fluorescent blue dye, to stain the cell nuclei. The results were analyzed using fluorescence microscopy, as seen in Figure 11b. The high level of green fluorescence in these experiments indicated the successful internalization of the hydrophobic drug.

## 4. Conclusions

In conclusion, the present study presents an innovative and straightforward method to create porous microgels through the judicious choice of gel precursors. This approach is achieved through a single-step procedure within a droplet microfluidics system, offering enhanced precision and control over the microgel formation process. By harnessing this technique, we successfully fabricated microgels characterized by their unique porous structures, which hold great potential for various applications. One of the most important features of our approach is the ability to adjust the porosity of these microgels by making specific changes to their composition. By modifying factors such as the concentration or ratio of components, we can introduce porosity within the microgel structure. Introducing porosity into microgels is a significant advancement, as it opens the door to various applications ranging from drug delivery to tissue engineering. The tailored structural characteristics of these porous microgels ensure efficient drug loading. As a result, the work reported here presents a streamlined approach to fabricating these microgels and underscores the far-reaching implications of their adjustable porosity within materials science and biomedical engineering.

## Figures and Tables

**Table 1:** Properties of microgels with varying monomers. While P signifies porous, NP signifies non-porous.

Microgels	PEGMEMA Eq.	PEGDA Eq.	PEG 10kDa Eq.
P-PEGMEMA-PEGDA	1.3	1	0.1
NP-PEGMEMA-PEGDA	1.3	1	-
P-PEGDA	-	1	0.1
NP-PEGDA	-	1	-

## Data Availability

Not applicable.

## Supplementary Materials

The following supporting information can be downloaded at: https://www.mdpi.com/article/10.3390/mi14101969/s1, Figure S1: FT-IR spectra of the components of the curcumin–HP-*β*-CD complex inclusion. Figure S2: H-NMR spectrum of the components of the curcumin–HP-*β*-CD complex inclusion in DMSO-d6. (a) Curcumin, (b) HP-β-CD, and (c) Curc–HP-β-CD inclusion complex. Figure S3: Microscopy images of microgels after release in cell medium. Figure S4: (a) DPPH solutions introduced to the curcumin and curcumin–HP-*β*-CD complex, (b) UV spectroscopy of DPPH solution and DPPH radical scavenging.
