# Supplementary material for: Porous Microgels for Delivery of Curcumin: Microfluidics-Based Fabrication and Cytotoxicity Evaluation
Source: Micromachines (Basel). 2023 Oct 22;14(10):1969. doi: 10.3390/mi14101969 (PMC10609253; doi:10.3390/mi14101969)
Supplement: Supplementary file 1 [file micromachines-14-01969-s001.zip › micromachines-2667585-supplementary.pdf]

SUPPLEMENTARY INFORMATION

Porous Microgels for Delivery of Curcumin: Microfluidics-based Fabrication and Cytotoxicity Evaluation

Sinem Orbay, Rana Sanyal, and Amitav Sanyal

1. *Curcumin* + HP-*β-CD Complexation*

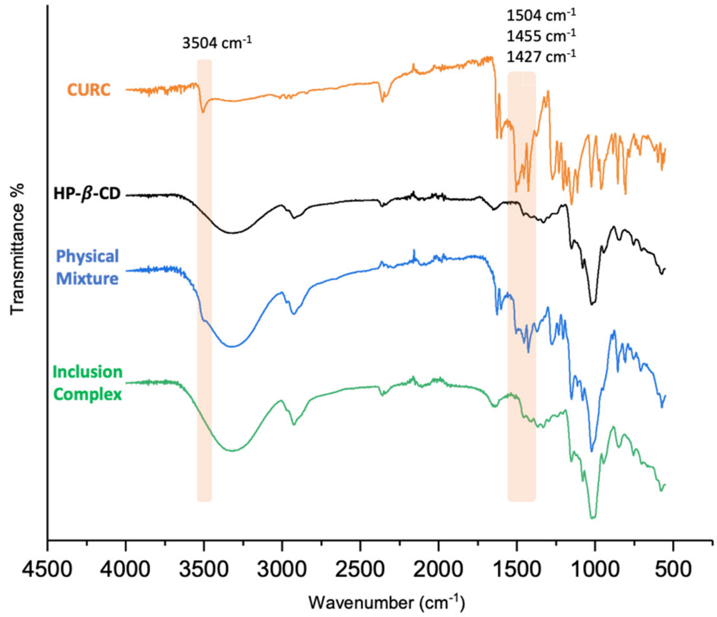

Figure S1. FT-IR spectra of the components of the Curcumin-HP-*β*-CD complex inclusion.

The figure provided displays FTIR spectra for CURC, HP-β-CD, a physical mixture of CURC and HP-β-CD, and the CURC/HP-β-CD inclusion complex. In the pure CURC spectrum, a weak band is observed at 3504 cm^{-1}, along with strong absorption bands at 1623, 1590, 1499, and 1424 cm^{-1}. Notably, the CURC spectrum differs from that of HP-β-CD (as shown in Figure).

The FTIR spectrum of the CURC and HP-β-CD physical mixture exhibits characteristic bands for both CUR and HP-β-CD, although with reduced intensity due to dilution. When comparing the

FTIR spectrum of the CUR/HP-β-CD inclusion complex with that of the physical mixture, a striking difference is observed. The absorption band at 3504 cm^{-1} completely disappears in the inclusion complex spectrum, and there is a significant decrease in band intensity at 1504, 1455, and 1427 cm^{-1} in comparison to their respective bands in the physical mixture. These observations strongly suggest the formation of an inclusion complex between CUR and HP-β-CD.

2. *H-NMR of Curcumin, HP-β-CD, and the inclusion in DMSO-d6*

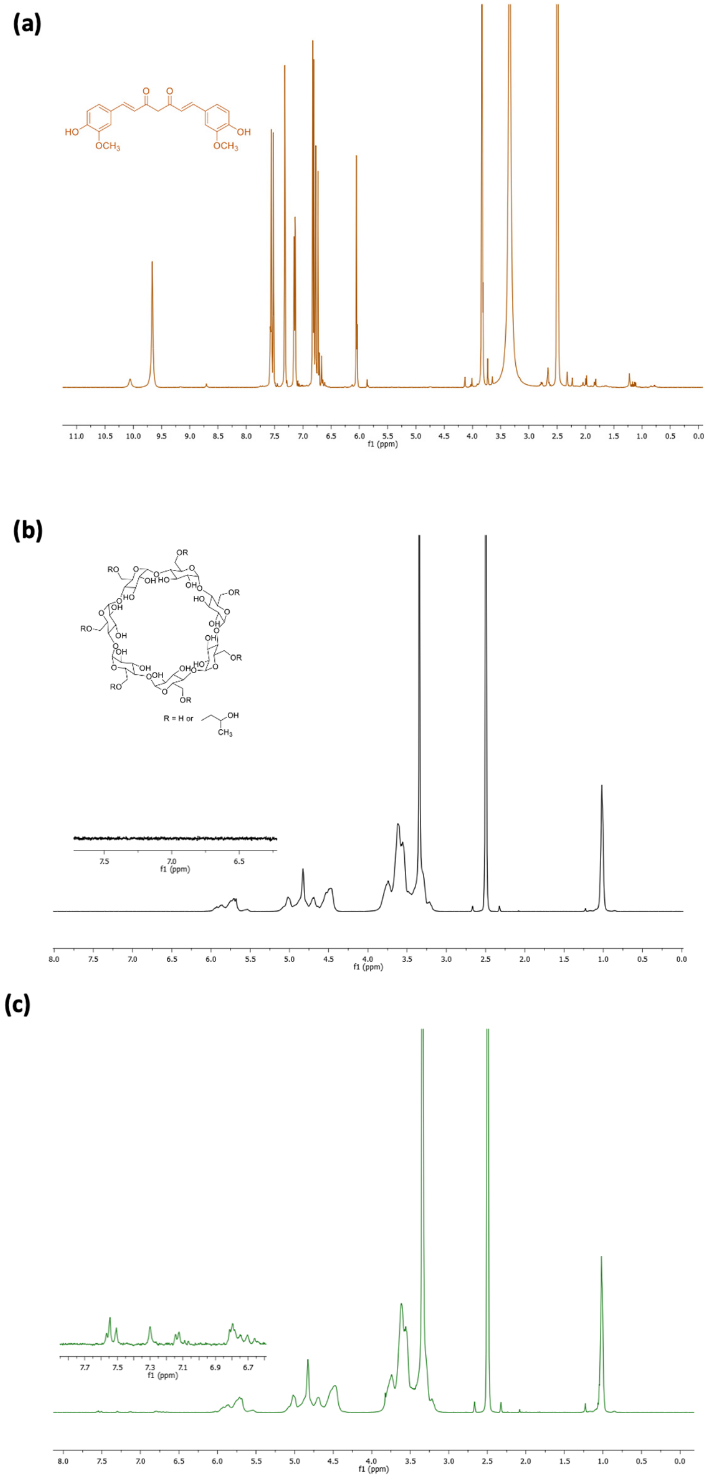

Figure S2. H-NMR spectrum of the components of the Curcumin/HP-*β*-CD complex inclusion in DMSO-d6. **(a)** Curcumin, **(b)** HP-β-CD and **(c)** Curc/ HP-β-CD inclusion complex.

3. Microscopy images after release in cell medium

Microscopy images after the release of curcumin from the microgels can be seen in Figure 3. Prior to the release process, the microgels exhibited bright fluorescence in the fluorescence microscopy images, attributable to the inherent fluorescence properties of curcumin. Following a 24-hour release period in a cell medium, the microgels were subjected to examination using both optical and fluorescence microscopy techniques. Optical images were employed to confirm the presence of the microgel particles, while the fluorescence images vividly illustrated the release of curcumin from these particles.

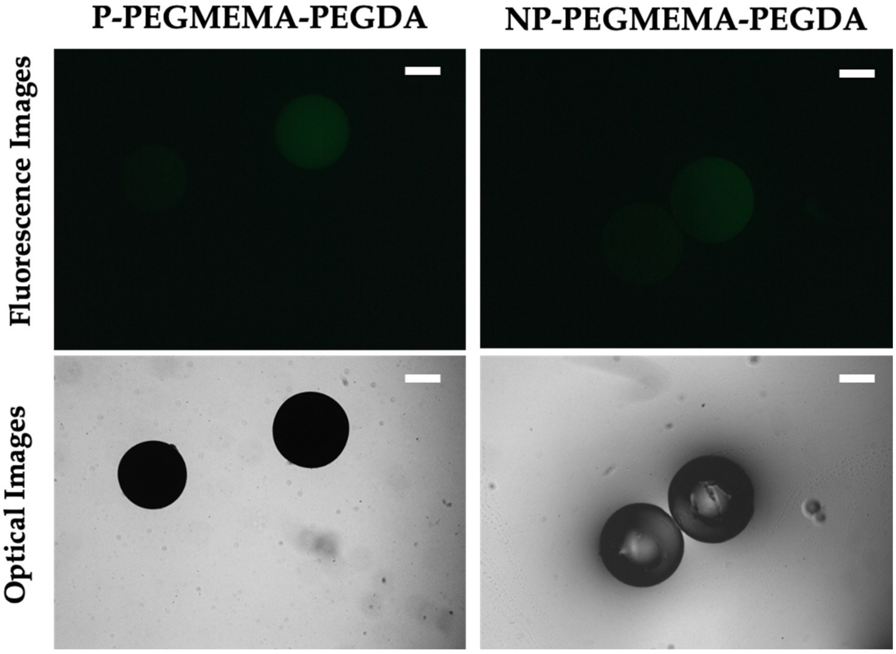

Figure S3. Microscopy images of microgels after release in cell medium (Scale 200 μm)

4. Antioxidant activity of Curcumin and Curcumin/HP-β-CD complex inclusion

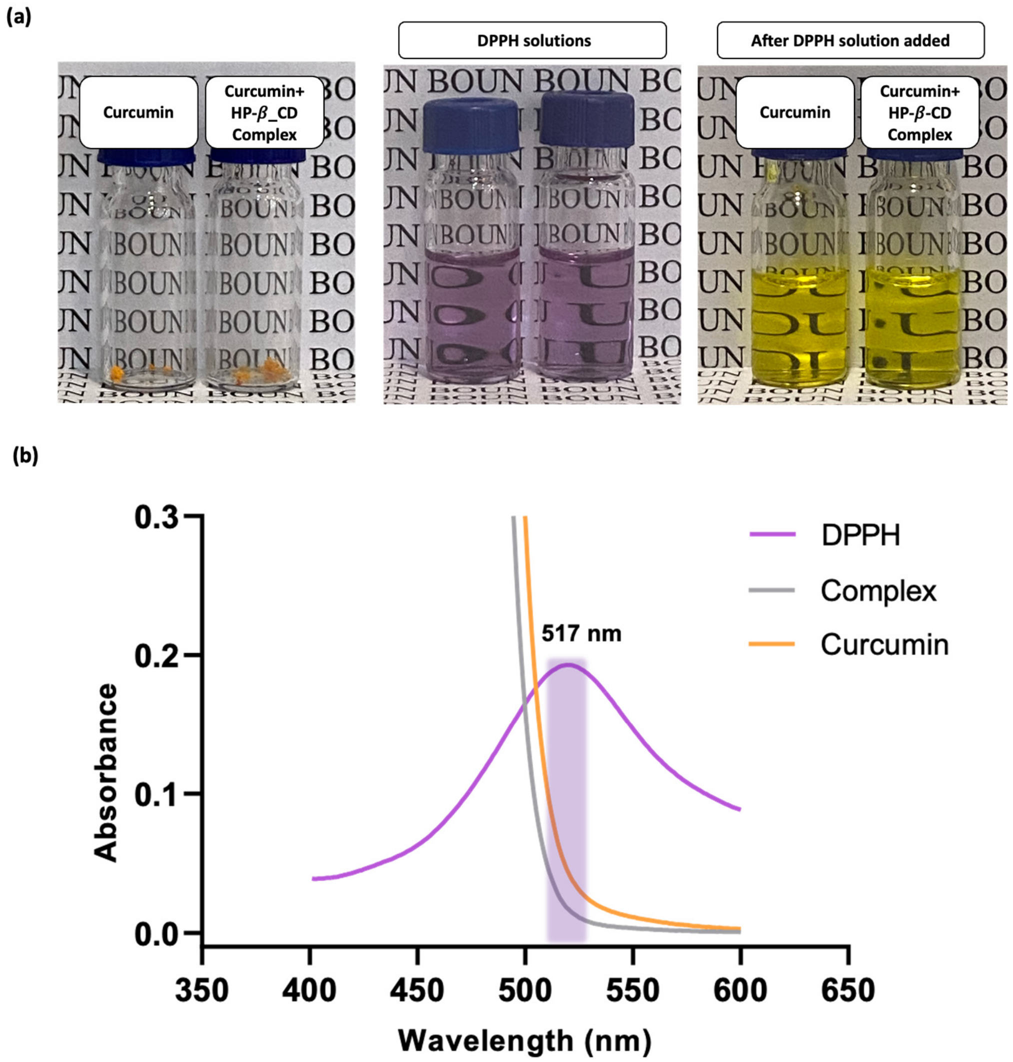

Figure S4. (a) DPPH solutions introduced to the Curcumin and Curcumin/HP-*β*-CD complex, **(b)** UV-vis spectra of DPPH solution and DPPH radical scavenging
